# Vitreous hemorrhage and Rhegmatogenous retinal detachment that developed after botulinum toxin injection to the extraocular muscle: case report

**DOI:** 10.1186/s12886-017-0649-2

**Published:** 2017-12-13

**Authors:** Dong Hyun Lee, Jinu Han, Sueng-Han Han, Sung Chul Lee, Min Kim

**Affiliations:** 1 Institute of Vision Research, Department of Ophthalmology, Severance Eye and ENT Hospital, Yonsei University College of Medicine, Seoul, South Korea; 2 Department of Ophthalmology, Gangnam Severance Hospital, Yonsei University College of Medicine, Seoul, South Korea

**Keywords:** Botulinum toxin, Retinal detachment, Vitreous hemorrhage, Strabismus

## Abstract

**Background:**

The authors report a case of a rare complication that occurred after botulinum toxin injection to the extraocular muscle, which was easily overlooked and successfully corrected by surgery.

**Case presentation:**

A 34-year-old female patient visited our clinic for diplopia and ocular motility disorder after removal of an epidermoid tumor of the brain. At her initial visit, her best-corrected visual acuity (BCVA) was 20/20 for both eyes. An alternate cover test showed 45 prism-diopter esotropia and 3 prism-diopter hypertropia in the right eye. Following 6 months of observation, the deviation of the strabismus did not improve, and botulinum toxin was injected into the right medial rectus (RMR). After 6 days, she visited our clinic with decreased visual acuity of her right eye. The BCVA was found to be 20/50 for her right eye. Funduscopic examination presented a retinal tear inferonasal to the optic disc with preretinal hemorrhage. Subretinal fluid nasal to the fovea was seen on optical coherence tomography (OCT). Barrier laser photocoagulation was done around the retinal tear; however, her visual acuity continued to decrease, and vitreous hemorrhage and subretinal fluid at the lesion did not improve. In addition, a newly developed epiretinal membrane was seen on OCT. An alternate cover test presented 30 prism-diopter right esotropia. 19 weeks after RMR botulinum toxin injection, she received pars plana vitrectomy, membranectomy, endolaser barrier photocoagulation, and intravitreal bevacizumab (Avastin®) injection. After 4 months, her visual acuity improved to 20/20, and only 4 prism-diopter of right hypertropia and 3 prism-diopter of exotropia were noted. Vitreous opacity and the epiretinal membrane were completely removed, as confirmed by funduscopic and examination.

**Conclusions:**

Sudden loss of vision after injection of botulinum toxin into the extraocular muscle may suggest a serious complication, and a prompt, thorough ophthalmic examination should be performed. If improvements are not observed, rapid surgical intervention is recommended to prevent additional complications.

**Electronic supplementary material:**

The online version of this article (10.1186/s12886-017-0649-2) contains supplementary material, which is available to authorized users.

## Background

Botulinum toxin is a widely used drug for the treatment of various ophthalmologic disease. In addition to cosmetic uses, botulinum toxin injection can be useful in the treatment of a variety of ocular muscle disorders, including strabismus [1], blepharospasm [2], upper eye lid retraction [3], entropion [4] and facial paralysis [5]. Botulinum toxin acts by blocking the release of acetylcholine from the synaptic cleft of the neuromuscular junction, thereby leading to chemical denervation of the muscle and reduced muscle activity. Further, during strabismus surgery, botulinum toxin acts to weaken the antagonist muscle and correct the deviation of the eyeball [6].

Rhegmatogenous retinal detachment (RRD) occurs when liquefied vitreous enters a previously created retinal hole or tear and causes detachment between the neurosensory retina and the retinal pigment epithelium (RPE). The detached retina should be reattached promptly to avoid severe vision loss. Surgical interventions to treat RRD include pars plana vitrectomy (PPV), scleral buckle (SB), PPV with a SB, and pneumatic retinopexy [7]. Proliferative vitreoretinopathy (PVR), a major complication of chronic RRD, is a common cause for the failure of surgical repairs. The prognosis of patients with PVR is poor because many cytokines derived from the vitreous trigger proliferation and migration of the RPE, leading to epithelial-mesenchymal transition and formation of a fibrous membrane that aggravates retinal detachment [8–11].

Although complications, including edema, bruising, pain, and headache, can occur as a result of botulinum toxin injection, most are self-limited and only last a short period [12]. However, severe complications, such as perforation of the globe, may develop inadvertently as a result.

The authors report a case of a young female who was diagnosed with RRD with PVR 6 days after botulinum toxin injection into the extraocular muscle. The patient was healed completely through surgical intervention.

## Case presentation

**Fig. 1:**
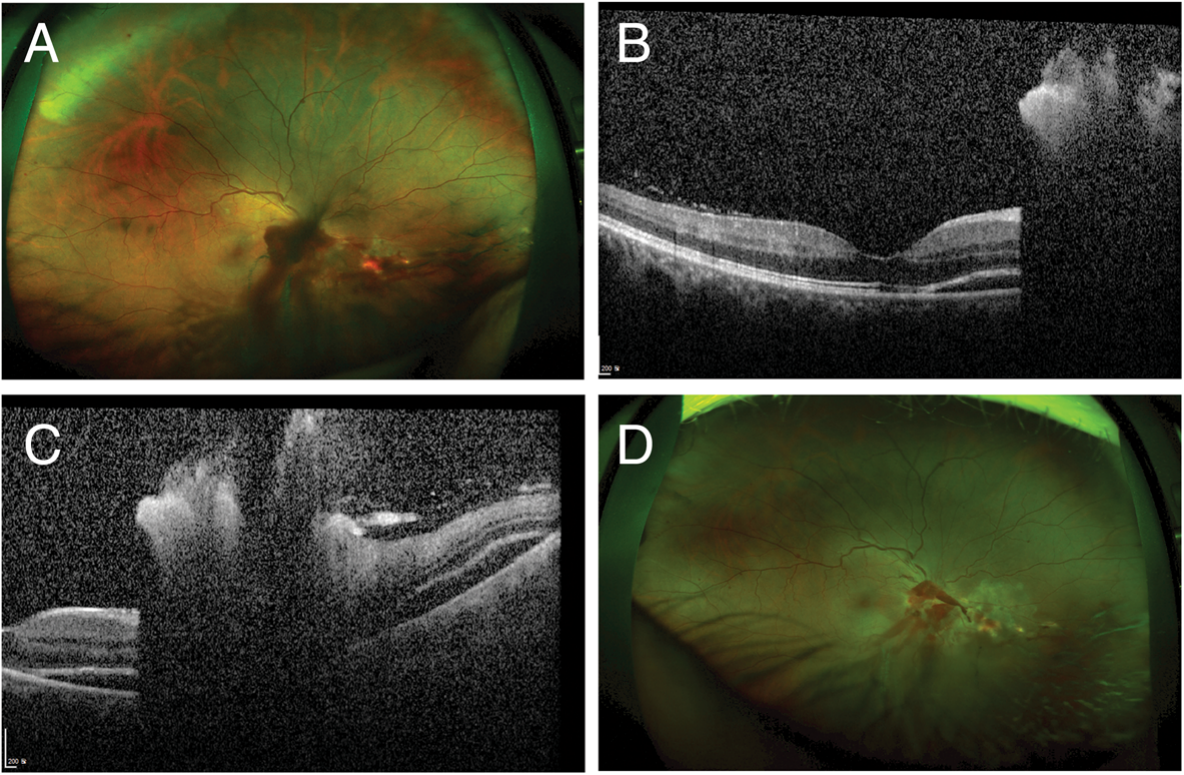
6 days after botulinum toxin injection to the right medial rectus muscle. **a** A wide-field color fundus photo shows a retinal tear inferonasal to the optic disc with preretinal hemorrhage nearly blocking the site. **b, c** SD-OCT B-scan images reveal subretinal fluid nasal to the fovea; however, the central fovea was intact. **d** One week after application of barrier laser photocoagulation around the retinal tear, the preretinal hemorrhage remained inferior to the optic disc

**Fig. 2:**
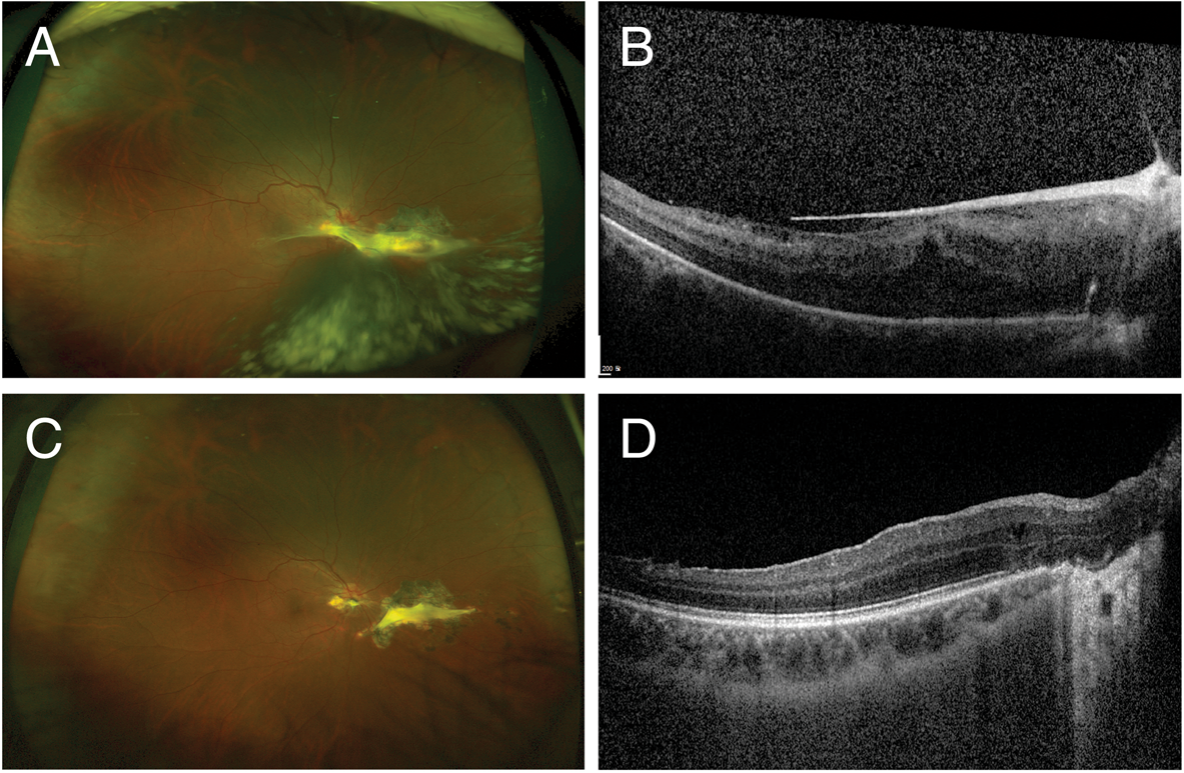
**a** 11 weeks after botulinum toxin injection. Note the fibrous membrane extending from the macula to the nasal mid-periphery and the vitreous hemorrhage that remained at the inferonasal quadrant. **b** A SD-OCT cross-sectional image reveals a thick epiretinal membrane corresponding to the fibrous membrane adherent to the central macula. **c** 18 weeks after PPV with visual acuity of 20/20. The scar change nasal to the optic disc remained, but the overall retina was remarkably flattened. **d** A SD-OCT B-scan image showed the flattened retina with minimal intraretinal cystic changes and a recovered photoreceptor layer

A 34-year-old female patient, with a history of laser epithelial keratomileusis, visited our clinic for diplopia and ocular motility disorder after removal of an epidermoid tumor at the prepontine cistern of the brain because of right 6th nerve palsy. At her initial visit, her best-corrected visual acuity (BCVA) was 20/20 for both eyes. An alternate cover test showed 45 prism-diopter esotropia and 3 prism-diopter hypertropia in the right eye. The patient was diagnosed with paralytic strabismus, and we planned to observe her for about two to three months to check changes in the amount of deviation. However, as the patient failed to visit outpatient clinic since then, further follow-up was unavailable. She revisited at 6 months after the development of her first symptom. Following 6 months of observation, angle of deviation did not improve, and 0.2 cm^3^ of botulinum toxin (2.5 international units) were injected into the right medial rectus (RMR). After 6 days, she visited our clinic complaining of decreased visual acuity of her right eye. The BCVA and intraocular pressure (IOP) for her right eye were found to be 20/50 and 15 mmHg, respectively. Based on funduscopic examination, a retinal hole was located 4 DD (disc diameter) inferonasally from the optic disc, and both preretinal and vitreous hemorrhages were present in front of the lesion (Fig. 1a). Optical coherence tomography (OCT) examination revealed subretinal fluid nasal to the fovea, but central fovea was attached (Fig. 1b, c). The patient was diagnosed with RRD with macula off, but because of small subretinal fluid and tiny retinal break, we decided to observe it first. Barrier laser photocoagulation around the retinal hole was performed (Fig. 1d). However, the patient’s visual acuity continued to decrease without improvement of vitreous hemorrhage, and at 12 weeks after botulinum toxin injection, a newly developed tractional membrane from macula to inferonasal periphery was noted, suggesting PVR development (Fig. 2a, b). An alternate cover test presented 30 prism-diopter right esotropia. 19 weeks following RMR botulinum toxin injection, she received PPV, membranectomy, peeling of the internal limiting membrane, and endolaser barrier photocoagulation. After 4 months, her visual acuity improved to 20/20, and only 4 prism-diopter of right hypertropia and 3 prism-diopter of exotropia were noted. Vitreous opacity and the epiretinal membrane were completely removed (Fig. 2c, d). Serious adverse complications, including endophthalmitis, retinal re-detachment, and increased IOP, were not observed.

## Discussion and conclusions

Although botulinum toxin is a relatively safe medication with few complications, some unexpected, adverse events can occur after injection. And a majority of those adverse events occurred immediately after or during procedure [13–15]. Liu M et al. reported a case in which the botulinum toxin was improperly injected into the vitreous cavity of a patient receiving treatment to the right medial rectus, resulting in increased IOP, decreased visual acuity, and retinal detachment [13]. The patient received topical and oral antibiotic treatment and spontaneously recovered without any further complications. In our case, however, the retinal detachment did not improve spontaneously, and the development of PVR necessitated surgical intervention.

Most studies have found intraocular botulinum toxin injection to be safe. Kutlut S et al. reported no difference in the retinal function of rabbit eyes that received intraocular botulinum toxin injection versus controls that received only intraocular saline injection, as confirmed by visual evoked potential and electroretinogram (ERG) test [16]. Likewise, Gatzioufas Z et al. reported IOP immediately increased after intravitreal botulinum toxin injection in normotensive rats; however, the density of retinal ganglion cells and immunostaining pattern of rhodopsin and retinal glial fibrillary acidic protein showed no difference between the group injected with botulinum toxin and the control group injected with balanced salt solution [17]. Pehere N et al. reported a case of unintentional injection of botulinum toxin into the eyeball that recovered without any complications other than a slight increase in intraocular pressure [18]. In our case, small amount of botulinum toxin, which might have been injected into the eyeball, did not affect the final visual acuity or intraocular pressure. To sum up, these reports suggest that, in most cases, botulinum toxin injected inadvertently into the eyeball does not appear to be toxic. However, periorbital botulinum toxin injection can cause mydriasis of the pupil, resulting in acute angle closure attack [19]. Further, spreading of the botulinum toxin to adjacent muscles, including the levator and inferior rectus muscles, can lead to the development of ptosis or vertical strabismus [20]. To solve these problems, peri- or retrobulbar anesthesia should be considered when electromyography (EMG) is not available [14]. By stretching the extraocular muscle slightly to make a free space between the extraocular muscle and the ocular surface [21], unintentional perforation of the globe can be prevented.

Although the procedure was done under EMG guidance, the patient underwent scleral perforation. Her axial length which was taken before PPV was 25.15 mm, which suggests that the patient might have had moderate myopia. Myopia is known to be a risk factor for scleral perforation in various surgical interventions, including strabismus surgery [22] and retro- or peribulbar anesthesia [23]. Therefore, we concluded that the risk factor was obscured due to past history of refractive surgery. Accordingly, when planning botulinum toxin injection into extraocular muscle, surgeon should be fully aware of patient’s refractive error as well as history of any kind of refractive surgery, in order to prevent inadvertent scleral perforation (Additional file 1 and Additional file 2).

In this case, the procedure was performed under EMG guidance, and no immediate problems were observed. However, after 6 days, the patient visited our clinic because of decreased visual acuity, and retinal detachment with preretinal hemorrhage was present. And the patient eventually had to undergo surgery to correct for the anatomical abnormality. Thus, the authors suggest that if a patient comes to the hospital presenting with a sudden loss of vision after botulinum toxin injection, a thorough ophthalmic examination, which includes a funduscopic examination, should be performed. If improvement is not observed, prompt surgical intervention must be executed to avoid more serious complications. To prevent such serious complications, surgeons should thoroughly review the patient’s past history and perform detailed examination to identify various risk factors, such as myopia and scleral scar, when planning botulinum toxin injection into extraocular muscle.

In conclusion, physicians should pay close attention to the uncommon complications that may arise during botulinum toxin injection, and respond properly in a suspicious situation with a variety of methods including either medical or surgical intervention. Moreover, knowing the patient’s risk factors before treatment helps to reduce such complications.

## Additional files

Additional file 1: Patient Perspective. This document has been confirmed to the patient who was the subject of this case report. (DOCX 17 kb)

Additional file 2: Timeline Picture of Our Case Report. This document is a simple diagram that shows a patient’s visit to our hospital, a series of tests that she had received, and healed course. (DOCX 46 kb)

## Abbreviations

BCVA: Best-corrected visual acuity
DD: Disc diameter
EMG: Electromyography
ERG: Electroretinogram
IOP: Intraocular pressure
OCT: Optical coherence tomography
PPV: Pars plana vitrectomy
PVR: Proliferative vitreoretinopathy
RMR: Right medial rectus
RPE: Retinal pigment epithelium
RRD: Rhegmatogenous retinal detachment
SB: Scleral buckle
