# Supplementary material for: Vitreous hemorrhage and Rhegmatogenous retinal detachment that developed after botulinum toxin injection to the extraocular muscle: case report
Source: BMC Ophthalmol. 2017 Dec 13;17:249. doi: 10.1186/s12886-017-0649-2 (PMC5729414; doi:10.1186/s12886-017-0649-2)
Supplement: Supplementary file 1 — Patient Perspective. This document has been confirmed to the patient who was the subject of this case report. (DOCX 17 kb) [file 12886_2017_649_MOESM1_ESM.docx]

Patient Perspective as follows:

“I suffered from complication after botulinum toxin injection for the treatment of strabismus. At first, I was worried visiting the clinic to evaluate blurred vision. After knowing that my eye was bleeding inside with retinal detachment, I was horrified. However, thanks to careful operation and care from Professor Kim, I can see much better now without pain or other complication.”
