# Supplementary material for: Vitreous hemorrhage and Rhegmatogenous retinal detachment that developed after botulinum toxin injection to the extraocular muscle: case report
Source: BMC Ophthalmol. 2017 Dec 13;17:249. doi: 10.1186/s12886-017-0649-2 (PMC5729414; doi:10.1186/s12886-017-0649-2)
Supplement: Supplementary file 2 — Timeline Picture of Our Case Report. This document is a simple diagram that shows a patient’s visit to our hospital, a series of tests that she had received, and healed course. (DOCX 46 kb) [file 12886_2017_649_MOESM2_ESM.docx]

**Timeline Picture of Our Case Report**

**Current Illness**

Decreased visual acuity of right eye

**6 days after RMR**

**botulinum toxin injection**

**34-year-old female patients**

**Past history** LASEK, removal of epidermoid tumor at the prepontine cistern

**Symptoms** diplopia and ocular motility disorder (45∆ RET, 3∆ RHT)

**Diagnosis** paralytic strabismus due to 6^th^ nerve palsy, right

**Interventions** observation at first → botulinum toxin injection into RMR

**Physical Examination**

**BCVA** 20/50

**IOP** 15mmHg

**Funduscopy**

retinal hole 4DD inferonasally from optic disc

preretinal and vitreous hemorrhage

**Diagnostic Evaluations**

**OCT** subretinal fluid nasal to the fovea

(central fovea was attached)

**Diagnosis**

Macula-off rhegmatogenous retinal detachment

**Initial Treatment**

Observation due to tiny retinal

break and small subretinal fluid

**6 days after RMR**

**botulinum toxin injection**

**Abbreviations**

**LASEK** laser epithelial keratomileusis

**RET** right esotropia

**RHT** right hypertropia

**RMR** right medial rectus

**BCVA** best-corrected visual acuity

**IOP** intraocular pressure

**OCT** optical coherence tomography

**DD** disc diameter

**PVR** proliferative vitreoretinopathy

**PPV** pars plana vitrectomy

**XT** exotropia

**ERM** epiretinal membrane

**4 months after PPV**

**Resolution of This Episode of Care**

**Final Follow-up**

**BCVA** 20/20

**EOM** 4∆ RHT, 3∆ XT

**Funduscopy** completely removed

vitreous opacity, ERM

**Ongoing Interventions**

PPV, membranectomy, peeling

of the internal limiting

membrane, endolaser barrier

photocoagulation

**19 weeks after RMR**

**botulinum toxin injection**

**12 weeks after RMR**

**botulinum toxin injection**

**Follow-up**

Newly developed tractional

membrane, suggesting PVR
